# Deep Learning-Based Child Handwritten Arabic Character Recognition and Handwriting Discrimination

**DOI:** 10.3390/s23156774

**Published:** 2023-07-28

**Authors:** Maram Saleh Alwagdani, Emad Sami Jaha

**Affiliations:** Department of Computer Science, Faculty of Computing and Information Technology, King Abdulaziz University, Jeddah 21589, Saudi Arabia; ejaha@kau.edu.sa

**Keywords:** child handwriting, handwritten character recognition, writer-group classification, convolutional neural network, deep learning, machine learning

## Abstract

Handwritten Arabic character recognition has received increasing research interest in recent years. However, as of yet, the majority of the existing handwriting recognition systems have only focused on adult handwriting. In contrast, there have not been many studies conducted on child handwriting, nor has it been regarded as a major research issue yet. Compared to adults’ handwriting, children’s handwriting is more challenging since it often has lower quality, higher variation, and larger distortions. Furthermore, most of these designed and currently used systems for adult data have not been trained or tested for child data recognition purposes or applications. This paper presents a new convolution neural network (CNN) model for recognizing children’s handwritten isolated Arabic letters. Several experiments are conducted here to investigate and analyze the influence when training the model with different datasets of children, adults, and both to measure and compare performance in recognizing children’s handwritten characters and discriminating their handwriting from adult handwriting. In addition, a number of supplementary features are proposed based on empirical study and observations and are combined with CNN-extracted features to augment the child and adult writer-group classification. Lastly, the performance of the extracted deep and supplementary features is evaluated and compared using different classifiers, comprising Softmax, support vector machine (SVM), k-nearest neighbor (KNN), and random forest (RF), as well as different dataset combinations from Hijja for child data and AHCD for adult data. Our findings highlight that the training strategy is crucial, and the inclusion of adult data is influential in achieving an increased accuracy of up to around 93% in child handwritten character recognition. Moreover, the fusion of the proposed supplementary features with the deep features attains an improved performance in child handwriting discrimination by up to around 94%.

## 1. Introduction

Despite significant advances in technology, the textual compositions of many people are still handwritten [1]. Thus, using automated recognition techniques for handwritten data in many applications is crucial. These techniques convert handwritten data (e.g., texts, words, characters, or digits) into corresponding digital representations, which can be accurately processed offline, such as scanned handwritten documents, or online, such as handwriting data input via electronic pen tip [2,3,4]. Developing automatic handwriting recognition systems is a difficult task in computer vision due to the wide variety of handwriting sizes and styles, besides the characteristics of the language to be recognized [4]. Handwritten character recognition is one of the most challenging research fields in document image processing. Most investigations in this field have been conducted on different languages (e.g., English, French, and Chinese), but only a little work has been conducted on other languages like Arabic [3].

In recent years, handwritten Arabic character recognition has gained considerable research interest. This is due to the importance of the Arabic language, which is considered one of the five most widely spoken languages worldwide and used for reading and writing by hundreds of millions of people from hundreds of nations [5]. However, it is considered a challenging task in pattern recognition and computer vision, as it still requires significant effort to construct generalized systems capable of handling various recognition problems and achieving highly feasible accuracy [6]. These challenges are due to the unique characteristics of the Arabic script, e.g., cursive nature, the existence of diacritics and dots, diagonal strokes, different alternative character shapes in the middle of words, and many characteristics [2,3,5]. Moreover, there are high diversities in handwriting styles across individuals; even at the individual level, a person’s handwriting may change significantly or slightly every time, which may make it difficult for a system to recognize the letters from their own handwriting [4].

The majority of research on handwritten Arabic character recognition has focused on adult handwriting, as the findings have revealed the effectiveness of their systems in achieving accuracy rates of up to 99% using deep learning and machine learning techniques [7,8,9,10,11,12,13,14,15]. Furthermore, a few researchers have recently focused on children’s handwriting data for recognizing Arabic letters due to its great significance for many applications and different purposes [4,16,17,18,19,20]. Employing character recognition capabilities in child-related applications such as education [21], interactive learning, physical or mental health assessment, or other possible practical purposes is critical for many future research areas. However, it poses a further challenge due to many differences between the nature of children’s and adults’ handwriting in several different aspects, including generally being of lesser quality, having more variances, and having more considerable distortions [16].

Handwritten Arabic character recognition technologies have evolved rapidly and achieved progress dramatically using different algorithms, such as support vector machines (SVMs), k-nearest neighbor (KNN), artificial neural networks (ANNs), and, later, convolutional neural networks (CNNs). CNNs have recently outperformed machine learning (ML) techniques that require manually generated features, while CNNs automatically detect and extract distinctive and representative features from the analyzed images [18]. Furthermore, building handwriting recognition hybrid systems using CNNs as a feature extractor and ML algorithms as a classifier has yielded effective results in several handwritten Arabic character datasets [15,18].

In this paper, we develop a novel CNN architecture for recognizing children’s handwritten isolated Arabic characters using the Hijja dataset [4] to compose a child data subset for testing. As previously stated, most recognition systems nowadays were neither trained nor tested on children’s handwriting character datasets. However, they were exclusively on adult datasets, although character recognition accuracy may be significantly improved by carefully considering the strategy in selecting the training dataset on which the model is to be trained. Therefore, we investigate and analyze the effect of training the suggested model using different datasets of handwritten Arabic characters by either children, adults, or both to assess and compare the fluctuations in model performance in recognizing children’s handwritten characters. Moreover, we use three popular machine learning SVM, KNN, and RF techniques as classifiers to assess the automatically derived features from the suggested trained CNN-based deep learning model and compare their performance variation; then, we observe how well these models perform in the classification process compared with the Softmax classifier.

For writer-group classification, Shin et al. [22] proposed a machine learning-based method to automatically classify individuals as adults or children based on their handwritten data, including Japanese scripts and drawn patterns. To the best of our knowledge, no similar research has focused on differentiating between children’s and adults’ handwriting for Arabic characters. Establishing this capability in this research could open new horizons for other research fields serving multiple purposes, such as fraud or forgery detection and prevention, recognizing and discriminating handwriting more accurately and working on improving skills, comprehending similarities and differences in ways of writing, and further estimating age groups. Finally, after analyzing handwriting, we propose some appropriate supplementary features that can be used along with the extracted deep features of the proposed CNN model to improve the accuracy of child and adult writer-group classification.

The main contributions of this study can be summarized as follows:

- Developing an effective CNN model for recognizing children’s handwritten Arabic characters.
- Investigating and analyzing the effect on child handwritten Arabic character recognition performance when training the proposed CNN model on a variety of datasets that either belong to children, adults, or both.
- Examining the capability of the suggested CNN model to classify the writers of Arabic characters into two writer groups, either children or adults.
- Suggesting some supplementary features that contribute to distinguishing between children’s and adults’ handwriting and augment the performance of the suggested CNN model.
- Extended performance analysis, evaluation, and comparison of the extracted deep features learned by the proposed CNN model and the proposed supplementary features using SVM, KNN, RF, and Softmax classifiers.

The rest of this paper is organized as follows: Section 2 discusses the previous studies on handwritten Arabic character recognition for adults and children data samples. Section 3 describes the used datasets and the proposed research methodology. The experimental work is demonstrated in Section 4, and the findings are provided in Section 5. Section 6 discusses the results and compares the proposed approach to other approaches from the literature. Finally, Section 7 concludes the proposed research work alongside a few ideas for future works.

## 2. Related Work

In this section, the previous work in the literature is reviewed, presenting various approaches using machine learning and deep learning techniques for adults’ and children’s handwritten Arabic character recognition. Most of the latest previous studies relevant to our work mainly focused on proposing different approaches to solve this challenging task using CNN-based models.

### 2.1. Handwritten Arabic Character Recognition for Adult Writers

Most researchers have focused on adult handwriting in Arabic character recognition. In 2017, El-Sawy et al. [7] developed a novel CNN model that was trained and tested on their own dataset, AHCD, which contains 16,800 handwritten Arabic characters collected from 60 persons aged between 19 and 40 years and divided into 28 classes, where their model achieved an accuracy of 94.9%. Another research by Younis [8] introduced a deep model using CNN to recognize handwritten Arabic letters, and it was improved by applying multiple optimization strategies to avoid overfitting. The results demonstrated that their model could classify letters using two datasets, AIA9k and AHCD, achieving 94.8% and 97.6% accuracy, respectively. In 2021, another new handwritten Arabic character dataset named HMBD was introduced by Balaha et al. [9]. They also suggested two CNN-based architectures known as HMB1 and HMB2. They investigated the effect of changing the complexity of these architectures using overfitting reduction strategies on various datasets, including HMBD, AIA9k, and CMATER, to increase recognition accuracy. The uniform weight initializer and the AdaDelta optimizer scored the highest accuracies, where the performance was improved via data augmentation using the HMB1 model, achieving the top overall performance of 90.7%, 98.4%, and 97.3% on AIA9k, HMBD, and CMATER datasets, respectively.

In [10], De Sousa suggested VGG12 and REGU deep models for recognizing handwritten Arabic letters and numbers. Both models were trained twice, once with data augmentation and once without. Then, an ensemble of the four models was created by averaging the predictions of each model. The highest accuracy of their ensemble model was 98.42% for AHCD and 99.47% for MADbase. Boufenar et al. [11] also built a DCNN model similar to Alexnet architecture. They investigated the role of preprocessing data samples in enhancing their model performance using three learning strategies: training the model from scratch, utilizing a transfer-learning technique, and fine-tuning the CNN. Overall, their experimental findings revealed that the first technique outperformed the others, either way, with and without preprocessing, achieving an average of 100% and 99.98% accuracy on OIHACDB-40 and AHCD, respectively. Moreover, Ullah et al. [12] investigated the dropout technique’s effect on their built CNN model. They noticed a considerable difference in performance when the model was trained with and without dropout, indicating that dropout regularization could effectively prevent model overfitting. The model reported a test accuracy of 96.78% on the AHCD dataset using dropout. Alyahya et al. [13] studied how the ResNet-18 architecture could be effective in recognizing handwritten Arabic characters. They suggested four ensemble models: the first two were the original ResNet-18 and the updated ResNet-18, using one fully connected layer with or without a dropout layer. The last two models were the original ResNet-18 and the updated ResNet18, but they included two fully connected layers with or without a dropout layer. The original ResNet-18 model achieved the highest test score of 98.30% from other ensemble models on the AHCD dataset. In [14], a CNN model was developed to recognize Arabic letters written by hand. The model was trained and tested using an AHCD dataset. Their experiment has shown that the suggested method achieved a recognition rate of 97.2%. Meanwhile, once data augmentation techniques were used, their model’s accuracy rose to 97.7%. Ali et al. [15] designed a CNN-based SVM model with a dropout technique utilizing two deep neural networks and evaluated it on various datasets, including AHDB, AHCD, HACDB, and IFN/ENIT, for recognizing handwritten Arabic letters. The authors reported improved performance of the suggested model compared to previous models created for the same domain by obtaining the accuracies of 99%, 99.71%, 99.85%, and 98.58% on AHDB, AHCD, HACDB, and IFN/ENIT, respectively. Table 1 summarizes these handwritten Arabic character recognition studies using adults’ data.

### 2.2. Handwritten Arabic Character Recognition for Child Writers

A few efforts have been made to address the issue of children’s Arabic handwriting recognition. In 2020, unlike earlier research, Altwaijry et al. [4] concentrated on recognizing Arabic letters for children’s writing. They collected a new dataset named Hijja, consisting of 47,434 disconnected and connected Arabic characters written by children aged 7 to 12 years. They also developed a functional CNN-based model to study and evaluate its performance on their dataset. They compared the performance of their model with the model suggested in El-Sawy’s paper [7] on both datasets, Hijja and AHCD. According to the experiment findings, their model outperformed the other compared model, achieving an accuracy of 88% and 97% on the Hijja and AHCD datasets, respectively. Alkhateeb et al. [16] also proposed a deep learning-based system for recognizing handwritten Arabic letters using CNN and three separate datasets, AHCR, AHCD, and Hijja, to validate the proposed system. Based on their experimental results, the suggested approach achieved accuracies of 89.8%, 95.4%, and 92.5% on the AHCR, AHCD, and Hijja datasets, respectively. Another study proposed by Nayef et al. [17] discussed using CNN models to recognize handwritten Arabic characters with an improved Leaky-ReLU activation function. To evaluate the performance of their compared models, they used four datasets, AHCD, HIJJA, and MNIST, in addition to their own dataset containing 38,100 handwritten Arabic characters, categorized into 28 classes that were collected from elementary school students in grades one to three. The proposed CNN model with Leaky-ReLU optimization outperformed the other compared model of [8] with an accuracy of 99%, 95%, and 90% on AHCD, their dataset, and Hijja, respectively.

Alrobah et al. [18] employed a different approach, merging CNN deep-learning models for feature extraction with SVM and XGBoost machine-learning models for classification to build a hybrid model. They used the two CNN architectures presented in [9], namely HMB1 and HMB2. The study attained an accuracy of 96.3% using the HMB1 model and the SVM classifier on the Hijja dataset, highlighting their hybrid model’s efficiency. In 2022, Wagaa et al. [19] presented a new CNN architecture that achieved 98.48% and 91.24% accuracies on the AHCD and Hijja datasets, respectively, by applying rotation and shifting data augmentation techniques and using the Nadam optimizer. They also investigated the impact of mixing the two AHCD and Hijja datasets of handwritten Arabic characters in varying proportions on the model’s performance during the training and testing phases using different data augmentation approaches. Their results showed that using the Nadam optimizer together with rotation and shifting data augmentation techniques gave their highest test accuracy of 98.32% among other choices when mixed with 80% of AHCD and 20% of Hijja for training along with 20% of AHCD and 10% of Hijja for testing. Bouchriha et al. [20] also presented a novel CNN model for recognizing handwritten Arabic characters. They focused on unique characteristics of Arabic text, particularly the difference in the shape of letters according to their location in the word, and by using the Hijja dataset, they attained an accuracy of 95%. Table 2 summarizes these handwritten Arabic character recognition studies on children’s data.

## 3. Proposed Methodology

**Figure 1:**
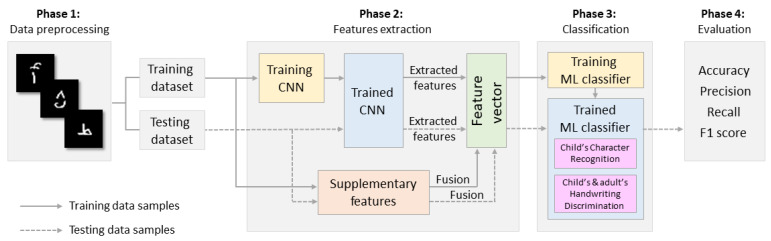
Overview of the framework of the proposed methodology.

In this study, we conducted two different tasks, handwritten character recognition and writer-group classification. Figure 1 shows the framework designed to achieve the suggested approach for recognizing children’s handwritten Arabic characters and classifying them into a child or an adult writer group. The proposed approach is divided into four phases: data preprocessing, feature extraction using CNN and other supplementary features, classification using three additional popular ML-based classifiers, and evaluation of the results and model performance using standard assessment measurement techniques. The following subsections provide more information on each of these four stages.

### 3.1. Data Preprocessing Phase

Data preprocessing is an important prior step meant to facilitate the extraction of significant features and improve classification accuracy. This section describes the datasets used to conduct the experimental work and how they were preprocessed using several methods.

#### 3.1.1. Datasets Description

This study uses two publicly available datasets of handwritten Arabic characters to conduct all experiments: Hijja dataset for child writers and the Arabic handwritten characters dataset (AHCD) for adult writers. The Hijja dataset [4] comprises 47,434 letter samples of size 32 × 32 written by 591 children aged 7 to 12 years. It has 108 subclasses arranged into 29 main classes, 28 classes for Arabic letters, and one more class for the “Hamza” character (ء). Each of the 28 classes contains up to four additional subclasses, categorized into connected (beginning, middle, and end of a word) and disconnected characters. Moreover, it was divided into 80% (37,933 samples) for training and 20% (9501 samples) for testing. Note that we only used the disconnected characters totaling 12,355 character samples for conducting the experimental work here. We divided them by the same proportion into two groups, 80% (9884 characters) for training and 20% (2471 characters) for testing.

The AHCD dataset [7] contains 16,800 character samples of size 32 × 32 written by 60 people aged 19 to 40 years, and it includes 28 classes for isolated (disconnected) Arabic characters. Each participant wrote each of the 28 characters ten times, from the character “Alf” (أ) to “Yaa” (ي). It was similarly divided into 80% for training with 13,440 samples (480 per class) and 20% for testing with 3360 samples (120 per class). Table 3 statistically describes the used child and adult datasets.

#### 3.1.2. Character Image Preprocessing

As mentioned above, we used all images in the Hijja dataset that only contain the character in its separate (disconnected) form. The preprocessing stage has included a number of procedures that help the proposed system achieve the highest possible accuracy. Firstly, these images were converted into grayscale images and then inverted to set the foreground as bright pixels and the background as dark pixels. Secondly, because some of the grayscale inverted images were too low-contrast and blurry, the contrast was adjusted to increase the intensity values of the foreground components and reduce the pixel values of the background to appear as dark as possible. After that, the brightness was raised by 2%. Thirdly, after empirically testing different image threshold values, the resulting pixels were thresholded by considering all values less than 90 as background pixels and resetting their values to zero. Finally, the foreground pixels were centered by drawing a rectangle around the character pixels and then cropped, after which zeros were added around the character to be the size 32 × 32.

For the AHCD dataset, the same thresholding was applied to all images and the characters were then centered. Moreover, the Hijja dataset has a different number of images for each class, which may negatively impact the efficiency of adequately training our deep model on all classes, especially in the comparison between different training strategies using child, adult, and both datasets. To solve this problem, we just increased the number of samples for each class in the Hijja training dataset using different data augmentation methods to match the number of samples for each class in the AHCD training dataset, which is 480, resulting in a sum of 13,440 character samples in the new augmented Hijja training dataset. The augmentation techniques used were zoom range, height shift range, and width shift range, all of which are equal to 0.1, and a rotation range of 5.

In addition, we combined both the Hijja and AHCD training and testing datasets to create a new dataset consisting of 26,880 characters for training and 5831 characters for testing. The combined training dataset was used in training both tasks of character recognition and writer-group classification, while the combined testing dataset was only used for probing the second task of writer-group classification. It is worth noting that, for writer-group classification, all images were further converted into binary images, unlike for character recognition using grayscale images.

### 3.2. Feature Extraction Phase

In this phase, the proposed features for handwritten character images and the classification process are extracted. This section explains the suggested CNN architecture and supplementary features in detail.

#### 3.2.1. Proposed CNN Architecture

CNNs have been proven successful and effective in recognizing handwritten characters [23]. A CNN is a multi-layered hierarchical model composed of convolution, pooling, and fully connected layers (FCLs). The purpose of convolution layers is to extract essential features from input images and generate feature maps using several filters. Pooling layers are used to minimize the dimensions of feature maps and to retrain the most critical features. Eventually, FCLs receive the high-level features from the preceding layers as input (formed as flat feature vectors) and yield several output classes, each with a value that indicates the class probability [23].

**Figure 2:**
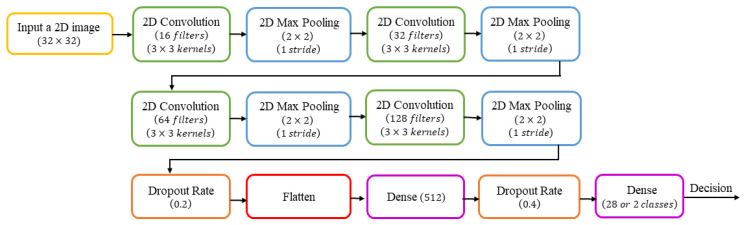
The proposed CNN architecture.

As shown in Figure 2, the suggested CNN model to extract features has ten layers, comprising four convolution layers, four max-pooling layers, and two fully connected layers. The input is a grayscale image for the child’s character recognition task and a binary image for the child’s handwriting discrimination task from the adult’s handwriting as a writer’s group, both images with a size of 32 × 32. All convolution layers use a 3 × 3 kernel, one stride, padding equal to the same input size, and a ReLU activation function that converts x (an input feature value) less than zero to zero, as defined in Equation (1).

(1) $$ReLU\;\left(x\right)=max\;(0,\;x)$$

The number of filters used in each convolution layer varies, such that the first convolution has 16 filters, the second has 32 filters, the third has 64 filters, and the fourth has 128 filters. Each convolution layer is followed by a max-pooling layer with a size of 2 × 2 and a stride of 1. A dropout rate of 0.2 is used after all convolution and max-pooling layers. Moreover, there are two dense layers in the last two fully connected layers. The first contains 512 neurons with a ReLU activation. In contrast, the second has 28 neurons for the character recognition task and two for the writer-group classification task with a Softmax activation, as defined in Equation (2), where $x_{i}$ is the output feature vector from CNN, *e* is a mathematical constant known as Euler’s number, and *N* is the number of output classes. After the first dense layer, a dropout rate of 0.4 was applied.

(2) $$Softmax\left(x_{i}\right)=\frac{e^{x_{i}}}{\sum_{k=1}^{N}e^{x_{k}}}$$

#### 3.2.2. Proposed Supplementary Features

The purpose of these features is to supplement the CNN-based features and improve the discrimination accuracy between children’s and adults’ handwriting. In this study, we used a histogram of oriented gradient (HOG)-based features and other statistical-based features as supplementary features to help distinguish between child and adult writers.

- Histogram of Oriented Gradient (HOG)-based Features

An HOG generates descriptive features for an object’s shape and appearance in an image by calculating gradients distribution or contour directions [24]. We used an HOG to extract features of the distinctive shape aspects of handwritten characters to distinguish children’s writing typical style from that of adults. To extract HOG features, the gradients for each pixel in the image were first computed in both the vertical and horizontal directions using the following Equations (3) and (4):

(3) $$d_{x}=I\left(x+1,\;y\right)-I(x,y)$$

(4) $$d_{y}=I\left(x,\;y+1\right)-I(x,y)$$

where $d_{x}$ and $d_{y}$ represent the horizontal and vertical gradient directions, and I(x,y) is the pixel value at (x,y). Hence, the gradient magnitude, |d|, and orientation, θ, were then calculated by Equations (5) and (6):

(5) $$|d|=\sqrt{d_{x}^{2}+d_{y}^{2}}$$

(6) $$\theta (x,y)={tan}^{-1}\frac{d_{y}}{d_{x}}$$

In the next step, the gradient image was divided into small cells of 8 × 8 pixels to calculate the histogram of gradient direction for each pixel inside the cell and place them into a nine-bin histogram. These histograms were then combined to represent HOG features. For better results, these histograms were then normalized by taking overlapping 3 × 3 blocks and applying L2-Hys normalization. Finally, the gradient histograms inside each cell between each block were then added together to obtain the final HOG feature vector of size 1 × 324.

- Statistical-Based Features

**Figure 3:**
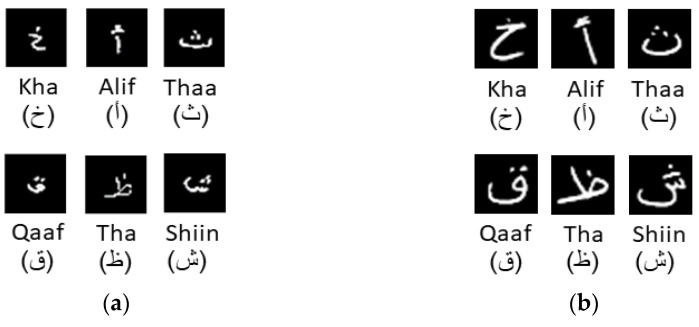
Some preprocessed Hijja and AHCD character data samples: (**a**) Child writers’ samples; (**b**) Adult writers’ samples.

We proposed some statistical-based features that assisted in differentiating between the handwriting of children and adults after analyzing their character data samples. Figure 3 shows the differences between adult and children’s handwriting for some characters such as Kha (خ), Alif (أ), Thaa (ث), Qaaf (ق), Tha (ظ), and Shiin (ش). Statistical features are based on the analysis of the spatial distribution of pixels and basic dimensions of a character sample [25]. The total of these features is twelve, as illustrated in Table 4, which are divided into two main groups as follows:

1. Ratio of Height to Width:

This feature depends on the main dimensions of a character, where the ratio of height h to width w (F1) is calculated for the bounding box of an unnormalized character sample (only the main body of the character, no “hamza” or “dots”) [25,26]. This feature is useful in differentiating between the sizes of letters written by children and adults since most letters written by children usually have common sizing characteristics, which can be utilized for differentiating them from those written by adults, and vice versa.

2. Ratios of Pixel Distribution:

These features depend on the spatial distribution of pixels in an image. We derived eleven features through the distribution of foreground fg (white) pixels and background bg (black) pixels for the bounding box of an unnormalized character sample. Firstly, we computed (F2) as the ratio of all foreground pixels to all background pixels for the whole character image [25]. Secondly, we divided the character image into four equal quadrants: upper-left (UL), upper-right (UR), bottom-left (BL), and bottom-right (BR) to calculate the ratio of the number of foreground pixels to the number of background pixels in each quarter (F3–F6). Finally, as inspired by [25], we computed the ratio of background pixels in each pairwise combination of the four quarters computed as $\left(\begin{array}{c} 4\\ 2 \end{array}\right)$, resulting in six features (F7–F12). These features are helpful for distinguishing pen strokes and font width between child and adult handwriting, as most of the children’s handwriting was intermittent, pen-down, pen-up actions, and displayed hesitancy, and it was somewhat light, while the adults’ handwriting was mostly uninterrupted and bold, indicating more confidence, convenience, and consistency.

### 3.3. Classification Phase

**Figure 4:**
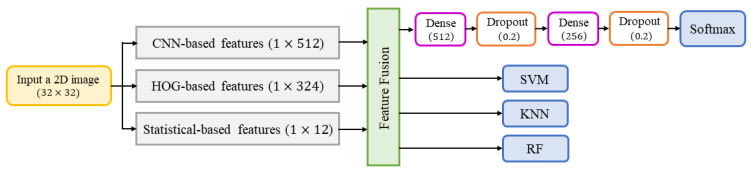
The FFNN-based feature fusion model for the writer-group classification task.

After the CNN model was trained and tested, we used it as a major feature extractor by replacing the final output FCL (Softmax classifier) with three well-known ML-based SVM, KNN, and RF classifiers for performance variation measurement and comparison purposes across all experiments. The feature vector obtained from the trained CNN consists of 512 features when trained on child data, adult data, and both for the children’s character recognition. For the writer-group classification task, we used the feature fusion method to supplement CNN-extracted features with statistical-based features, with HOG-based features, and with both. The feature vector obtained from the statistical-based feature extractor constitutes twelve features, while the one obtained from the HOG-based feature extractor comprises 324 features. All these extracted features were normalized to range from 0 to 1 using min-max normalization. The fused feature vector was initially trained and evaluated using the Softmax classifier by constructing a feed-forward neural network (FFNN) with six layers. The first layer is the input layer showing the number of features in each feature vector, while the remaining layers are illustrated in Figure 4. All these extracted features were then trained by the ML classifiers. After training, the trained ML classifiers were used for testing in the classification phase for children’s handwritten character recognition and children and adult handwriting discrimination with/without supplementary features.

#### 3.3.1. Support Vector Machine (SVM) Classifier

SVM is an effective supervised learning technique used for classification and regression tasks. It works on training data examples as plotted points in a high-dimensional feature space. The classification process is then performed by finding an optimal hyperplane that separates between classes correctly when achieving the maximum possible margin between them [1,27,28]. The nascent SVM performance significantly relies on the three primary hyperparameters: kernel function, regularization (usually defined as *C*), and *gamma* [15]. In this work, we used a nonlinear SVM classifier that can be defined as shown in Equation (7).

(7) $$f\left(x\right)=\sum_{i=1}^{l}w_{i}\Phi_{i}(x)+b$$

where $\Phi \left(x\right)$ represents a feature map and w refers to the corresponding weights. Φ means transform x input vector from input space into a higher dimensional feature space using kernel functions. Kernel functions have two main parameters: *C* and *gamma*. We examined here multiple nonlinear SVM kernel functions with several values of *C* and *gamma* to find the optimal values that yield the best possible classification accuracy.

#### 3.3.2. K-Nearest Neighbor (KNN) Classifier

KNN is the simplest supervised learning classifier, requiring no previous intensive training process or probabilistic classification. It works by finding *k* nearest samples and their class labels in the training dataset to predict the class of a new sample in the testing dataset. The classification process is performed by measuring the distance between feature vectors of training and testing samples in feature space. The *k*-nearest samples with their class labels are then retrieved to choose the predominant class label as a class for the test sample [29]. In this work, we tested different distance metrics with different odd k numbers, where the distance measures used in KNN are Euclidean and Manhattan distances, which can be defined as given in Equations (8) and (9), respectively [30].

(8) $$d\left(x,y\right)=\sqrt{\sum_{i=1}^{n}(x_{i}-y_{i}{)}^{2}}$$

(9) $$d\left(x,y\right)=\sum_{i=1}^{n}\left|x_{i}-y_{i}\right|$$

where x and y are the feature vectors in the feature space, and $x_{i}$ and $y_{i}$ refer to their *i*-th feature of the total n features.

#### 3.3.3. Random Forest (RF) Classifier

RF is an ensemble machine learning algorithm used for classification and regression problems [31]. It is composed of multiple decision trees that are generated in parallel using a subset of randomly selected training data samples, each of which works as an independent classifier. Their predictions are then aggregated to determine the final outcome by calculating the majority vote for the results of each output decision tree. RF enables fast learning even with high-dimensional features. Moreover, the random selection of training data makes it robust against noise [22]. This work tested different numbers of trees and their maximum depth.

### 3.4. Evaluation Phase

The overall performance of the proposed model was evaluated using the *accuracy*, *precision*, *recall*, and *F*1-*score* metrics inferred via the four distributions, true positive (*TP*), false negative (*FP*), true negative (*TN*), and false negative (*FN*), as follows:

- *Accuracy* (*A*) is the ratio of correctly predicted characters to the total of all predicted characters. Equation (10) shows the accuracy evaluation metric.
  (10) $$Accuracy=\frac{TP+TN}{TP+FP+FN+TN}$$
- *Precision* (*P*) is the ratio of correctly predicted positive characters to the total number of correctly and incorrectly predicted positive characters. Equation (11) shows the precision classification rate.
  (11) $$Precision=\frac{TP}{TP+FP}$$
- *Recall* (*R*) is the ratio of correctly predicted positive characters to the total number of positive characters, calculated using Equation (12).
  (12) $$Recall=\frac{TP}{TP+FN}$$
- *F*1*-score* (*F*1) combines the recall and precision measures, as shown in Equation (13).
  (13) $$\;F1-score=\frac{2(Recall\times Precision)}{Recall\times Precision}$$

## 4. Experiments

This section describes the environment used, experimental setup, and design of experiments with implementation details, hyperparameter tuning, and data augmentation.

### 4.1. Experimental Setup

All experiments were conducted using the Google Colab environment. In addition, several open source Python libraries were used, such as Kares to build and train the CNN model, Scikit-learn to address ML classifiers and print evaluation measurement tools, CSV to read Excel data files, TensorFlow to implement and evaluate the CNN model, and others.

### 4.2. Experiments Design

In this research, we conducted five experiments with different scenarios. The first three experiments are related to testing the proposed CNN model in recognizing children’s handwritten Arabic letter data (Hijja) by training the model on children’s data (Hijja), on adult data (AHCD), and on both types of data (combined Hijja and AHCD). The last two experiments are associated with discriminating between adult and child handwriting of Arabic letters by training and testing the model on both data samples (combined Hijja and AHCD) with and without the proposed supplementary features. The extracted features by the CNN and supplementary features are trained and evaluated using Softmax, SVM, KNN, and RF classifiers. Table 5 briefly describes the objective of each experiment. The three datasets used for experimental work were prepared and rearranged by dividing them into 80% for training and 20% for testing for all classifiers. To tune the CNN model’s hyperparameters, the training dataset was divided into 60% for training and 20% for validation. Table 6 shows for each experiment the number of images and the image type of each of the training, validation, and test datasets for tuning the proposed CNN model’s hyperparameters.

### 4.3. Hyperparameters Tuning and Data Augmentation

To tune the proposed CNN model’s hyperparameters, we examined three different optimizers and three weight initializers in all experiments using the validation dataset to find the optimal hyperparameters for the training dataset in order to make the model generalized and as not overfitted as possible. The examined optimizers are Adam, Nadam, and RMSProp, while the weight initializers are Normal, Uniform, and He Normal. Nadam optimizer and He Normal weight initializer are used to optimize our model since they gave better results than the others. In addition, categorical cross-entropy was used to calculate the loss for the child’s character recognition and binary cross-entropy for the writer-group classification, where accuracy was assigned as the metric. The model was also trained using a batch size equal to 80 and an epoch number set to 100. Moreover, we used the ReduceonLRPPlateau approach that periodically reduces the learning rate in the Kares library, beginning from 0.001 until 0.00001 when multiplied by a factor equal to 0.1. For the FFNN model, it was also trained using 80 batch sizes and 100 epochs, with the Nadam optimizer and the binary cross-entropy.

We set the following hyperparameters for the SVM classifier: *C* = (1, 10, 100, 1000), kernel = [‘poly’, ‘sigmoid’, ‘rbf’], and *gamma* = (0.01, 0.001, 0.0001), whereas for the KNN classifier we set the following: *k* = (5, 7, 9, 11), weights = [‘distance’], and metric = [‘Euclidean’, ‘Manhattan’]. We also set the hyperparameters for the RF classifier as n_estimators = (50, 100, 200, 300, 400) and max_depth = (5, 10, 15, 20, 25, 30). We tuned the hyperparameters of these classifiers using the grid search method and then determined the optimal hyperparameters that provide the highest possible classification accuracy. Finally, we used the same data augmentation techniques applied to the Hijja training dataset to be balanced in training the CNN model by increasing the overall size of the Hijja and AHCD datasets, with a view to overcome the overfitting problem and improve the model’s performance.

## 5. Results

The results obtained from the five conducted experiments are reported in this section to evaluate and compare the proposed model’s performance using different classifiers for recognizing children’s handwritten Arabic characters and distinguishing between child and adult handwriting. It is worth noting that the data split for all five experiments using different classifiers was 80% for training and 20% for testing.

**Figure 5:**
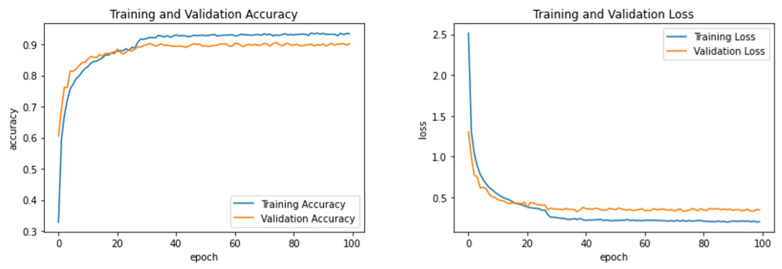
Learning accuracy and loss performance of Experiment 1.

Experiment 1 was conducted to show how the proposed model performed after being trained and tested on the Hijja dataset alone. The results of this experiment are presented in Table 7. Furthermore, the accuracy and loss curves of the training and validation are shown in Figure 5. The model achieved the best performance with an accuracy of 91.95% using the SVM classifier with radial basis function (SVM-RBF) kernel values set to (*C* = 100 and *gamma* = 0.001). The RF classifier reported the second-highest accuracy at 91.87%, while Softmax and KNN achieved the lowest performance compared to the others. It is worth noting that the hyperparameter of the KNN was set to Manhattan distance and *k* = 5, and the RF was set to n_estimators = 300 and max_depth = 30.

**Figure 6:**
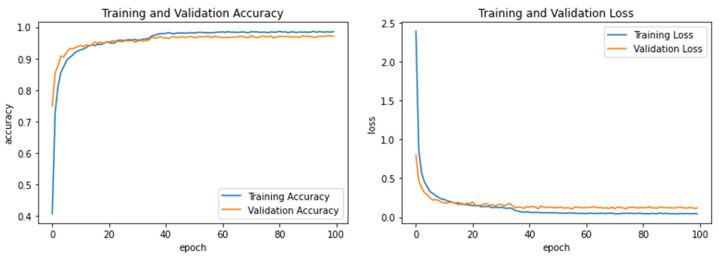
Learning accuracy and loss performance of Experiment 2.

Experiment 2 investigated the effect of training the proposed model using the adult handwriting dataset (AHCD) alone on testing the child handwriting dataset (Hijja). Table 8 shows the results obtained in Experiment 2, and Figure 6 displays its accuracy and loss curves over the training epochs. Here, the highest accuracy reported in this experiment was 80.17%, achieved by the SVM classifier, while KNN, RF, and Softmax received lower accuracies of 79.24%, 79.16, and 78.67%, respectively. Noting that the hyperparameter of the SVM-RBF kernel was set to *C* = 10 and *gamma* = 0.01, KNN was set to Manhattan distance, with *k* = 9, and RF was set to n_estimators = 200 and max_depth = 20.

**Figure 7:**
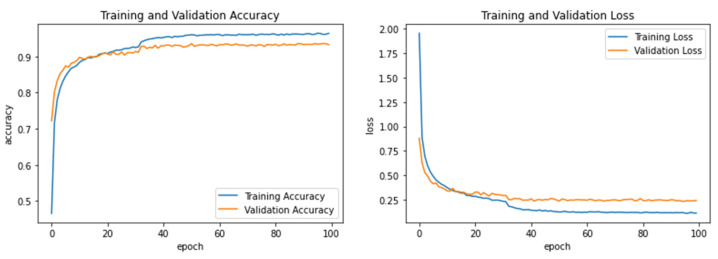
Learning accuracy and loss performance of Experiment 3.

Experiment 3 was carried out to see whether the proposed model could improve recognition accuracy when trained on both child and adult data samples (combined Hijja and AHCD) to recognize the Hijja testing dataset. Table 9 summarizes all the recognition results of the Hijja testing dataset when child and adult datasets were combined during the training phase. Also, the accuracy and loss curves are shown in Figure 7. Interestingly, this experiment achieved a higher accuracy of 92.96% for both SVM and Softmax and 92.72% for RF and 92.47% for KNN than the prior two experiments trained only on either the child or adult dataset in isolation. It is worth noting that hyperparameter of the SVM-RBF kernel was set to *C* = 10 and *gamma* = 0.01, KNN was set to Manhattan distance and *k* = 9, and RF was set to n_estimators = 400 and max_depth = 25. Table 10 summarizes and compares the performance results of the three experiments along with their average performance of the different classifiers used.

**Figure 8:**
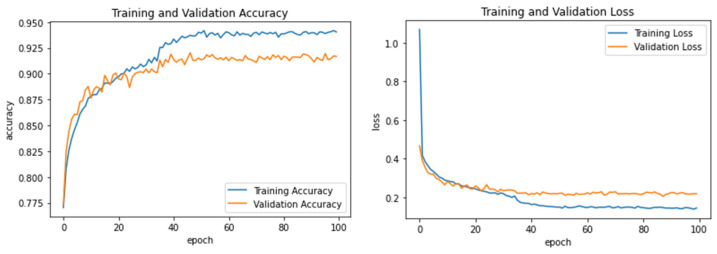
Learning accuracy and loss performance of Experiment 4.

Experiments 4 and 5 assessed how well the suggested model could classify writers based on their handwriting into two groups: a child writer and an adult writer, with/without supplementary features. In Experiment 4, we trained and tested the CNN model without using supplementary features. Table 11 shows the model’s writer-group classification performance. Moreover, Figure 8 illustrates the learning accuracy and loss performance for Experiment 4. The RF classifier received the best accuracy of 90.41%, where n_estimators was set to *C* = 200 and max_depth = 30. On the other hand, the SVM, KNN, and Softmax classifiers achieved a lower accuracy of 89.85%, 89.74%, and 88.24%, respectively, where the SVM-RBF kernel values were set to *C* = 100 and *gamma* = 0.01, and the hyperparameters of KNN were Euclidean distance and *k* = 11.

**Figure 9:**
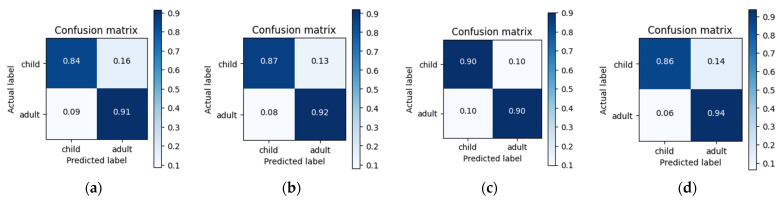
Confusion matrix depicting the results of Experiment 4 using three different classifiers: (**a**) Softmax classifier, (**b**) SVM classifier; (**c**) KNN classifier; (**d**) RF classifier.

The results of Experiment 4 were also analyzed and validated using the confusion matrix, as shown in Figure 9. Hence, we observed that the RF classifier, shown in Figure 9d, outperformed the Softmax, SVM, and KNN classifiers by achieving 94% accuracy for accurate adult classification and only 6% of adults were misclassified as children. Nevertheless, the best child classification accuracy was 90% using the KNN classifier, as shown in Figure 9c, whereas only 10% of the child samples were misclassified as adult ones.

For Experiment 5, we combined the CNN-extracted deep features as follows: first, with statistical-based features (SF) resulting in a 524-dimensional feature vector; second, with HOG-based features resulting in an 836-dimensional feature vector; third, with both SF and HOG features resulting in an 848-dimensional feature vector. The results reported in Table 12 show that, when all extracted features from CNN, SF, and HOG were fused, we received the highest performance for all classifiers, for all evaluation metrics, with the highest achieved accuracies of 93.98%, 92.11%, 92.06%, and 91.00% for Softmax, KNN, SVM, and RF, respectively. When CNN and HOG features were combined, they achieved the second-highest accuracy scores ranging from 90.94% to 93.88, whereas the lowest accuracies were scored by CNN and SF fusion ranging from 89.88% to 91.92%. It is worth noting that, in all three fusion cases, the Softmax classifier was superior, by all means, in writer-group classification performance over KNN, RF, and SVM. Subsequently, compared to the results of Experiment 4, by utilizing the fusion of CNN deep features with the proposed supplementary SF and HOG features, the classification performance of discriminating between adult and child handwriting was significantly improved by up to 5.74%, 5.48, 2.24%, and 3.86% for accuracy, precision, recall, and F1-score, respectively, where the average scores of these four metrics were also enhanced by up to 3.11%, 2.01%, 1.71%, and 1.84%, respectively. The hyperparameter of the SVM-RBF kernel was set to *C* = 100 and *gamma* = 0.01 for each combination of features fusion, the KNN distances were set as Euclidean with *k* = 7, Manhattan with *k* = 9, and Manhattan with *k* = 11, and the RF was set to n_estimators = 200, 300, and 200 with max_depth = 30, respectively.

## 6. Discussion and Comparison

### 6.1. Discussion of the Results

In this work, extensive experiments were conducted to investigate how the proposed methodology can improve performance in the more challenging task of children’s handwritten Arabic character recognition. The investigation was conducted by training the model on child data samples, adult data samples, and both. In addition, we evaluated the proposed approach’s capabilities in classifying writers of testing handwritten character samples as adults or children and how the proposed supplementary features can help improve the classification accuracy. Based on analysis of the results of Experiments 1 to 3, it can be observed, as in Table 10, that when the (child) Hijja and (adult) AHCD datasets were merged for the training of the proposed model, as in Experiment 3, to recognize child handwritten Arabic characters, achieving 92.78% average accuracy with about 1% higher score than the 91.78% average accuracy obtained by training the proposed model on the (child) Hijja dataset only, as in Experiment 1. Such an improvement in accuracy may be a result of providing the trained model with clearer and higher-quality supportive samples of the (adult) AHCD dataset during training, which enhanced the modeling process and increased the trained model’s ability to recognize more confusable children’s letter samples via balanced and non-overfitted learning as possible by combining both child and adult training data. Consequently, incorporating adult data alongside children’s data during the training phase improved the recognition accuracy of the (child) Hijja test datasets.

In contrast, when the same model was trained on the (adult) AHCD dataset alone and tested on the (child) Hijja dataset, as in Experiment 2, the recognition accuracy decreased noticeably, scoring a lower average accuracy of 79.31% compared to Experiment 1 and 3. The reason is that the (child) Hijja dataset is considered more complex and challenging than the (adult) AHCD dataset since it contains many letter samples that can be distorted, unclear, and low-quality, as we noticed and also stated in [16]. In other words, the model was trained using only adult samples that were fairly clear and more consistent in terms of the characteristics, shape, and size of the letter, and there were no notable distortions compared with the Hijja dataset of child samples. Due to this, the trained model could not recognize numerous confusable (child) Hijja data samples.

After analyzing the results and seeing the misclassified samples of Experiment 4, we observed that there were similarities between the handwriting of children and adults in terms of character and sizing characteristics, presence of distortions, pen stroke, and font width. Based on these similarities, some children’s data samples were classified as being written by adults because they were mostly closer to the common writing style of adults. On the contrary, some adult data samples were classified as being written by children due to the presence of some adult samples with a similar style to the common children’s writing style. Several factors led to such similarities between child and adult writing styles, such as growing age and educational level. The quality of a person’s handwriting improves with growing age, except for exceptionally aging or sick people. Also, the higher a person’s education level, the more likely it is that their handwriting will be better. However, for most of those grown people who resort to overusing technology devices rather than traditional paper-and-pen, over time, their handwriting may remain or become low-skilled, closer to children’s handwriting level. In Experiment 5, combining all the proposed supplementary features (SF and HOG) or only HOG with the CNN deep features contributed to increasing discrimination accuracy between child and adult handwriting by approximately 6% using the Softmax classifier. Generally, in Experiment 5, the classification accuracy was improved in all cases compared with Experiment 4 in various ratios, as shown in Table 12.

### 6.2. Comparison with Existing Works

We compared our suggested methodology with different related approaches from the literature that concentrated on children’s handwritten Arabic character recognition using the Hijja dataset. The comparison was made in terms of the target task, the methods used for feature extraction and classification, suggested supplementary (handcrafted) features, applied feature fusion technique, and the dataset used for training and testing the model. In these studies [4,16,17,20], researchers developed a new character recognition system using a CNN deep learning model, trained it, and tested it on children’s data samples. In [18], they designed a hybrid model by combining existing CNN models as feature extractors with SVM and XGBoost machine learning models as classifiers, which were trained and evaluated using the Hijja dataset. The hybrid model has outperformed other models using the SVM classifier. However, these studies did not investigate the effect of training their suggested models on the AHCD dataset (comprising adult data samples) or on a combination of the (child) Hijja and (adult) AHCD datasets to be eventually only focused and tested on the more challenging Hijja child data samples alone.

In [19], a novel CNN model was created, trained, and evaluated using children’s data. In addition, they studied how the use of data augmentation techniques affected the performance of recognition during the model training and testing when combining the two datasets, Hijja and AHCD, in various ratios. Nevertheless, they did not train their model on combined child and adult datasets in an equal proportion to exclusively be tested on the challenging child dataset. Finally, none of these studies or earlier studies addressed classifying handwritten Arabic letters into child or adult writer groups. Table 13 demonstrates different aspects of comparison between the suggested strategy and several methodologies used in earlier studies.

## 7. Conclusions

In this paper, several experiments were conducted for two tasks: handwritten character recognition and writer-group classification. First, we designed a CNN model for children’s handwritten Arabic character recognition. Then, the model was used to study the impact of the training process on various handwritten Arabic character datasets belonging to children, adults, or both in particularly recognizing letter samples written by children only. We concluded that, when the model was trained on both samples of children and adult data, we achieved the best performance and obtained the highest average accuracy of 92.78%, which is rather higher than the accuracy resulting from training the model on children’s data in isolation. Moreover, training the model on adult data alone, even though there are much higher-quality data compared to child data, had a negative effect on the model’s performance in recognizing children’s data.

The same model with necessary changes was also used to examine and assess its capability to differentiate between children’s and adults’ handwriting. As a result, it initially achieved an average classification accuracy of 89.28%, demonstrating after extended analysis that there could be considerably confusable similarities in writing style between adults and children. To confront such confusable similarities and improve the child handwriting discrimination performance and results, we suggested HOG-based and statistical-based supplementary features to supplement the deep features extracted from the CNN model. Amongst three proposed feature fusion approaches in Experiment 5, the approach combining CNN-based deep features with both statistical-based and HOG-based supplementary features augmented the model’s performance in distinguishing between child and adult handwriting using combined Hijja and AHCD for training and testing. It yielded the highest average accuracy of 92.29%, about 2.73% higher than the result obtained using only CNN features. In addition, we trained and tested all extracted features using Softmax, SVM, KNN, and RF classifiers, where SVM with the RBF kernel gave a higher accuracy than the Softmax classifier in the character recognition task. On the other hand, in the writer-group classification task, Softmax was the superior classifier among all, according to all performance evaluation measures.

For future work, this approach can be extended and used to recognize handwritten connected Arabic letters for children and propose further useful supplementary features that may contribute to improving character recognition accuracy. Moreover, the capability of this approach using some intentional mistakes can also be investigated and analyzed. Moreover, it can also be enforced in various practical applications to discriminate between children’s and adults’ handwriting through texts or words.

## Figures and Tables

**Table 1:** A summary of related work on handwritten Arabic character recognition for adult writers.

Ref.	Year	Feature Extractor	Classifier	Dataset	Type	Size	Accuracy
[7]	2017	CNN	Softmax	AHCD	Characters	16,800	94.9%
[8]	2017	CNN	Softmax	AIA9k AHCD	Characters Characters	9000 16,800	94.8% 97.6%
[10]	2018	CNN	Softmax	AHCD MADbase	Characters Digits	16,800 70,000	98.42% 99.47%
[11]	2018	CNN	Softmax	OIHACD AHCD	Characters Characters	30,000 16,800	100% 99.98%
[9]	2020	CNN	Softmax	HMBD AIA9k CMATER	Characters Characters Digits	54,115 9000 3000	90.7% 98.4% 97.3%
[13]	2020	CNN	Softmax	AHCD	Characters	16,800	98.30%
[14]	2021	CNN	Softmax	AHCD	Characters	16,800	97.7%
[15]	2021	CNN	SVM	AHDB AHCD HACDB IFN/ENIT	Words and Texts Characters Characters Words	15,084 16,800 6600 26,459	99% 99.71% 99.85% 98.58%
[12]	2022	CNN	Softmax	AHCD	Characters	16,800	96.78%

**Table 2:** A summary of related work on handwritten Arabic character recognition for child writers.

Ref.	Year	Feature Extractor	Classifier	Dataset	Type	Size	Accuracy
[4]	2020	CNN	Softmax	Hijja AHCD	Characters Characters	47,434 16,800	88% 97%
[16]	2020	CNN	Softmax	AHCR AHCD Hijja	Characters Characters Characters	28,000 16,800 47,434	89.8% 95.4% 92.5%
[17]	2021	CNN	Softmax	AHCD Proposed dataset Hijja MNIST	Characters Characters Characters Digits	16,800 38,100 47,434 70,000	99% 95.4% 90% 99%
[18]	2021	CNN	Softmax SVM XGBoost	Hijja	Characters	47,434	89% 96.3% 95.7%
[19]	2022	CNN	Softmax	AHCD Hijja	Characters Characters	16,800 47,434	98.48% 91.24%
[20]	2022	CNN	Softmax	Hijja	Characters	47,434	95%

**Table 3:** Description of the used datasets.

Dataset Characteristic	Hijja	AHCD
Number of writers	591	60
Total samples per character for each writer	1	10
Total character samples per writer	28	280
Total samples per character	400~500	600
Total isolated character samples	12,355	16,800
Category of writers	Children	Adults

**Table 4:** A summary of statistical-based features.

Feature	Formula	Feature	Formula	Feature	Formula
F1	h/w	F5	${\left(fg/bg\right)}_{BL}$	F9	${bg}_{UL}/{bg}_{BL}$
F2	${\left(fg/bg\right)}_{All}$	F6	${\left(fg/bg\right)}_{BR}$	F10	${bg}_{UR}/{bg}_{BR}$
F3	${\left(fg/bg\right)}_{UL}$	F7	${bg}_{UL}/{bg}_{UR}$	F11	${bg}_{UR}/{bg}_{BL}$
F4	${\left(fg/bg\right)}_{UR}$	F8	${bg}_{UL}/{bg}_{BR}$	F12	${bg}_{BL}/{bg}_{BR}$

**Table 5:** An overview of conducted experimental work.

Experiment No.	Task	Training Dataset	Testing Dataset
Experiment 1	Character Recognition	Hijja	Hijja
Experiment 2		AHCD	Hijja
Experiment 3		Combined Hijja and AHCD	Hijja
Experiment 4	Writer-Group Classification *without* Supplementary Features	Combined Hijja and AHCD	Combined Hijja and AHCD
Experiment 5	Writer-Group Classification *with* Supplementary Features	Combined Hijja and AHCD	Combined Hijja and AHCD

**Table 6:** Statistics of the used datasets.

Dataset	Training Dataset	Validation Dataset	Testing Dataset	Normalized Image Type
Hijja	10,752	2688	2471	Grayscale
AHCD	10,752	2688	2471	
Combined Hijja and AHCD	21,504	5376	2471	
Combined Hijja and AHCD	21,504	5376	5831	Binary

**Table 7:** Child character recognition results of Experiment 1, using Hijja for training and testing.

Classifier	*Accuracy*	*Precision*	*Recall*	*F*1-*Score*
Softmax	91.78%	91.87%	91.76%	91.76%
SVM	**91.95%**	**92.07%**	**91.91%**	**91.93%**
KNN	91.50%	91.62%	91.46%	91.47%
RF	91.87%	91.93%	91.82%	91.81%

Results in bold indicate the highest scores achieved among the different classifiers.

**Table 8:** Child character recognition results of Experiment 2, using AHCD for training and Hijja for testing.

Classifier	*Accuracy*	*Precision*	*Recall*	*F*1-*Score*
Softmax	78.67%	80.54%	78.66%	78.87%
SVM	**80.17%**	**81.87%**	**80.12%**	**80.28%**
KNN	79.24%	81.12%	79.21%	79.40%
RF	79.16%	80.62%	79.12%	79.15%

Results in bold indicate the highest scores achieved among the different classifiers.

**Table 9:** Child character recognition results of Experiment 3, using combined Hijja and AHCD for training and Hijja for testing.

Classifier	*Accuracy*	*Precision*	*Recall*	*F*1-*Score*
Softmax	**92.96%**	92.99%	**92.92%**	92.92%
SVM	**92.96%**	**93.14%**	92.91%	**92.94%**
KNN	92.47%	92.52%	92.44%	92.42%
RF	92.72%	92.81%	92.68%	92.69%

Results in bold indicate the highest scores achieved among the different classifiers.

**Table 10:** Aggregated child character recognition results and average performance of Experiments 1 to 3.

	Experiment 1		Experiment 2		Experiment 3	
Classifier	*Accuracy*	*F*1-*Score*	*Accuracy*	*F*1-*Score*	*Accuracy*	*F*1-*Score*
Softmax	91.78%	91.76%	78.67%	78.87%	**92.96%**	**92.92%**
SVM	91.95%	91.93%	80.17%	80.28%	**92.96%**	**92.94%**
KNN	91.50%	91.47%	79.24%	79.40%	**92.47%**	**92.42%**
RF	91.87%	91.81%	79.16%	79.15%	**92.72%**	**92.69%**
Average	91.78%	91.74%	79.31%	79.43%	**92.78%**	**92.74%**

Results in bold indicate the highest scores achieved among the different classifiers.

**Table 11:** Writer-group classification performance of Experiment 4, without supplementary features using combined Hijja and AHCD for training and testing.

Classifier	*Accuracy*	*Precision*	*Recall*	*F*1-*Score*
Softmax	88.24%	88.57%	91.37%	89.95%
SVM	89.85%	90.56%	91.96%	91.26%
KNN	89.74%	92.44%	89.52%	90.96%
RF	**90.41%**	**90.50%**	**89.82%**	**90.11%**
Average	89.56%	90.52%	90.67%	90.57%

Results in bold indicate the highest scores achieved among the different classifiers.

**Table 12:** Writer-group classification performance of Experiment 5, with supplementary features using combined Hijja and AHCD for training and testing.

	CNN + SF				CNN + HOG				CNN + SF + HOG			
Classifier	*A%*	*P%*	*R%*	*F1%*	*A%*	*P%*	*R%*	*F*1*%*	*A%*	*P%*	*R%*	*F*1*%*
Softmax	91.92	91.85	91.57	91.70	93.88	93.91	93.54	93.71	**93.98**	**94.05**	**93.61**	**93.81**
SVM	89.88	89.75	89.48	89.61	92.03	**91.762**	91.96	91.86	**92.06**	91.760	**92.08**	**91.90**
KNN	90.00	89.66	90.01	89.81	91.75	91.42	91.87	91.60	**92.11**	**91.77**	**92.29**	**91.98**
RF	90.14	90.17	89.59	89.84	90.94	91.30	90.18	90.61	**91.00**	**91.35**	**90.23**	**90.67**
Average	90.49	90.36	90.16	90.24	92.15	92.10	91.89	91.95	**92.29**	**92.23**	**92.05**	**92.09**

Results in bold indicate the highest scores achieved among the different classifiers.

**Table 13:** Comparison between our proposed methodology and current approaches in the literature.

Ref.	Task	Feature Extraction		Feature Fusion	Classification				Dataset Used	
		CNN	Handcrafted		Softmax	SVM	KNN	RF	Training	Testing
[4]	Character Recognition	√			√				Hijja	Hijja
[16]		√			√				Hijja	Hijja
[17]		√			√				Hijja	Hijja
[20]		√			√				Hijja	Hijja
[18]		√			√	√			Hijja	Hijja
[19]		√			√				Hijja	Hijja
		√			√				A mixture of Hijja and AHCD in a different ratio	A mixture of Hijja and AHCD in a different ratio
Our study	Character Recognition	√			√	√	√	√	Hijja	Hijja
		√			√	√	√	√	AHCD	Hijja
		√			√	√	√	√	A mixture of Hijja and AHCD in an equal ratio	Hijja
	Writer-Group Classification	√	√	√	√	√	√	√	Both Hijja and AHCD	Both Hijja and AHCD

## Data Availability

The datasets used in this article were Hijja and AHCD. For details, please refer to [4,7].
